# A wearable activity tracker intervention for promoting physical activity in adolescents with juvenile idiopathic arthritis: a pilot study

**DOI:** 10.1186/s12969-018-0282-5

**Published:** 2018-10-22

**Authors:** Liane D. Heale, Saunya Dover, Y. Ingrid Goh, Victoria A. Maksymiuk, Greg D. Wells, Brian M. Feldman

**Affiliations:** 1 Division of Rheumatology, The Hospital for Sick Children, 555 University Ave, Toronto, ON M5G 1X8 Canada; 2 Child Health Evaluative Sciences, The Hospital for Sick Children Research Institute, 686 Bay St, Toronto, ON M5G 0A4 Canada; 3 Department of Pediatrics and the Dalla Lana School of Public Health, University of Toronto, 155 College St., Toronto, ON M5T 3M7 Canada

**Keywords:** Physical activity, Juvenile idiopathic arthritis, Exercise, Health behaviors, Activity tracker, Adolescent

## Abstract

**Background:**

Children and adolescents with juvenile idiopathic arthritis (JIA) are less physically active than their healthy peers and are at high risk of missing out on the general health benefits of physical activity. Wearable activity trackers are a promising option for intervening in this population with potential advantages over traditional exercise prescriptions. The objectives of this study were to: (1) determine the feasibility of a wearable activity tracker intervention in adolescents with JIA; and (2) estimate the variability in response to a wearable activity tracker intervention on the physical activity levels of adolescents with JIA.

**Methods:**

Participants aged 12–18 years with JIA were recruited during their routine rheumatology clinic visits at a tertiary care hospital. Participants completed the 3-Day Physical Activity Recall self-reported questionnaire at baseline, 1 week and 5 week follow-up. At the 1 week follow up, participants were instructed to start wearing an activity tracker for 28 consecutive days. Participants completed a feasibility questionnaire at their end of study visit. Participant demographics, adherence rates and feasibility outcomes were summarized using descriptive statistics. The effect of wearing a tracker on moderate-to-vigorous physical activity (MVPA) and total metabolic equivalents (METs) per day were analyzed using a paired t-test.

**Results:**

Twenty-eight participants (74% female; median age 15.1, range 12.8–18.6) were included in the analysis. All of the participants were able to synchronize the activity tracker to a supported device, use the activity tracker correctly and complete the study measurements. On average, participants had activity logged on their smartphone application for 72% of the intervention period. The standard deviation of the change in mean METs/day was 12.148 and for mean MVPA blocks/day was 3.143 over the study period.

**Conclusion:**

Wrist worn activity tracking is a feasible intervention for adolescent patients with JIA. More research is needed to examine the effect of activity tracking on physical activity levels.

**Trial Registration:**

Not an applicable clinical device trial as per the criteria listed on ClinicalTrials.gov as the primary objective is feasibility.

## Background

Regular physical activity is associated with health benefits in all children. Higher levels of physical activity are associated with improved body composition [1], metabolic profile [2] and measures of bone health [3, 4]. Conversely, low levels of physical activity are associated with increased cardiovascular disease risk factors [4, 5]. In addition to physical health benefits, habitual physical activity is associated with improved measures of mental health including increased self-esteem, cognitive performance, academic achievement, and reduction in depression [4] and anxiety [6].

A 2011 report indicated that Canadian youth are not meeting Canadian Physical Activity Guideline recommendations for optimal health [7]. Similar results have also been reported in youth studies in the United States [8, 9] and globally [10]. For most measures of health, aerobic-based activities provide the greatest benefit [4]. For optimal bone health, high-impact weight bearing activities are required [4]. Currently, the Canadian Physician Activity Guidelines [11] and the WHO Global Recommendations on Physical Activity for Health [12] recommend that children and youth aged 5–17 accumulate at least 60 min of moderate-to-vigorous physical activity (MVPA) daily. They should also participate in vigorous-intensity activities and activities that strengthen muscle and bone at least 3 days per week. Unfortunately, less than 10% of Canadian youth are adhering to these recommendations [7].

Children and adolescents with juvenile idiopathic arthritis (JIA) are even less physically active than their healthy peers [13–15]. Using accelerometers to objectively measure physical activity, Bohr et al. reported lower levels of total activity, and MVPA when children and adolescents with JIA were compared with age and gender-matched controls [13]. Adolescents with JIA also have lower levels of sports participation [16]. These low levels of physical activity persist despite adequate disease control [13]. As a consequence, young people with JIA have reduced cardiorespiratory fitness [14, 15] and bone mineral density [17] when compared with age-matched controls.

The impact of physical activity on JIA disease course and symptomatology has not been established. Despite theoretical advantages of reducing loss of proteoglycans and cartilage damage, and optimizing bone mineral density, there is no established relationship between physical activity levels and disease activity or functional ability [14, 15, 18]. The relationship between physical activity and pain in JIA is also complex and results of studies have been conflicting [19].

While the effect of physical activity on disease activity is unknown, randomized controlled trials with exercise interventions in JIA were well tolerated and resulted in improvements in cardiovascular fitness [20, 21].

Considering the low levels of physical activity reported in the JIA population in childhood and the tendency for a precipitous decline in physical activity during adolescence [22], young people with JIA are at high risk of missing out on the general health benefits of physical activity. Because of this, physical activity interventions are critical for this vulnerable population.

Wearable activity trackers are a promising option for intervening in this population with potential advantages over traditional exercise prescriptions. Wearable technology is increasing in popularity and offers real-time feedback (in the form of progress towards an activity goal) that has the potential to motivate patients. The trackers also offer social platforms that could inspire healthy competition with peers. In addition, activity trackers can provide physicians with objective measurements of patients’ progress and response to medical therapies.

While a recent systematic review suggested that activity trackers have the potential to increase physical activity levels of healthy youth, it concluded that there is a paucity of research on the feasibility and effectiveness of activity trackers in this age group [23]. Furthermore, although some research has examined the feasibility of activity trackers in youth with chronic disease [24, 25], to the best of our knowledge there are no studies to date that have described their use in the adolescent JIA population.

Thus, the primary objectives of this study are to: (1) determine the feasibility of a wearable activity tracker (the Misfit Flash™) intervention in adolescents with JIA; and (2) estimate the variability in the effect size of an activity tracker intervention on the physical activity levels of adolescents with JIA, for use in planning a definitive trial. Exploratory objectives include determining the effects of an activity tracker on patient-reported health outcomes, pain, active joint count and physician global rating of disease activity.

## Methods

### Design

In this feasibility pilot, a single-group pre- and post-intervention study design was used to determine the feasibility of studying the effect of a wearable activity tracker on physical activity levels, patient reported health outcomes, pain, active joint count and physician global rating of disease activity. Participants completed study measurements at three time points: with a clinic visit at study enrollment; over the telephone and with online questionnaires 1 week after study enrollment and with a clinic visit after at least 28 days of wearing the activity tracker (Table 1).

**Table 1:** Overview of data collection at study visits

	Baseline	1 week	≥ 5 weeks
Demographics	X		
3DPAR	X	X	X
PROMIS® scales	X	X	X
Pain Scale	X		X
CHAQ	X		X
Joint Count	X		X
Physician Global Rating	X		X
Activity tracker start and stop		START	STOP

### Study subjects

Boys and girls aged 12–18 years who met ILAR classification criteria for JIA [26] were selected for inclusion in the study if (1) their disease status was considered stable by their rheumatologist; (2) they were unlikely to require modification to medication therapy for the duration of the study; and (3) they had access to a smart phone or tablet compatible with the activity tracker chosen for this study – the Misfit Flash™.

Patients were excluded from participation if they (1) had moderate or high disease activity based on 2011 American College of Rheumatology recommendations for the treatment of JIA [27]; (2) had changes to their JIA medications in the 3 months prior to study enrollment; (3) had significant cardiovascular, respiratory or metabolic comorbidity; and (4) were already using an activity tracker at the time of enrolment.

### Recruitment

Participants were recruited during their routine clinic visits in the general rheumatology clinics and medical day care unit at The Hospital for Sick Children. This study was approved by the Research Ethics Board at The Hospital for Sick Children. All participants provided written informed consent.

A convenience sample was used with enrollment of consecutive patients fulfilling study criteria. The planned sample size was 30 participants, a sufficient number for our feasibility aims.

### Measurements

#### Physical activity

The 3-Day Physical Activity Recall (3DPAR) was completed at all three study time points. The 3DPAR is a self-report instrument that was designed to capture habitual physical activity of adolescents and has been validated in subsequent studies in this age group [28, 29].

The 3DPAR instrument requires the recall of activity performed during each of the three previous days, beginning with the most recent day. Each day is segmented into 34 30-min time blocks (7:00 a.m. to midnight). The instrument provides a list of common activities grouped into the following categories: sleep/bathing, eating, work, after-school/spare time/hobbies, transportation, and physical activities/sports [30]. For each block of the day, participants were asked to report the main activity in which they participated during each 30-min time period. Participants also rated the relative intensity of each activity as light, moderate, hard, or very hard. A member of the research team facilitated completion of the questionnaire at all study time points.

Following completion of the questionnaire, each 30-min block was assigned a metabolic equivalent (MET) value based on the specific activity and level of intensity reported by the participant [30]. MET values were summed over each of the 3 days for a measure of total daily physical activity (METs/day). In addition, the number of 30-min blocks in which energy expenditure was estimated at three METs or greater (i.e., MVPA blocks) were summed for each individual day (MVPA blocks/day). Three-day averages for METs/day, MVPA blocks/day were then calculated.

#### Self-reported pain, fatigue and depressive symptoms

Participants completed the Patient Reported Outcomes Measurement Information System (PROMIS®) questionnaires at all study time points. The PROMIS® tool provides a measure of patient-reported health status for physical, mental and social well-being. Participants completed the short form tools in the following domains: (1) pain interference, (2) fatigue, and (3) depressive symptoms. Participants were asked to rank the frequency with which they experienced symptoms in the past 7 days. Scores for each symptom range from 0 – never experienced the symptom, to 4 – almost always experienced the symptom. A total score was calculated by taking the sum of the scores (0–4) for each item in that domain. There are 8 items in the pain interference and depressive symptoms domains; thus, scores ranged from 0 to 32. Total scores in the 10-item fatigue domain ranged from 0 to 40.

#### Clinical assessment

Participants had a clinical assessment at study enrollment and study completion. As part of routine follow up visits in the rheumatology clinic, physicians recorded the total number of active and swollen joints and provided a physician global rating of participant disease activity. Participants were asked to rate their pain in the past week on a 10-cm visual analog scale from 0 (no pain), to 10 (very severe pain). They completed the Child Health Assessment Questionnaire (CHAQ) [31] as a self-report measure of their functional impairment.

#### Dropout

We tracked participant dropout as part of our feasibility aims. At the conclusion of the study, we asked participants if they had any illness or injury that interfered with their ability to participate in physical activity during the study.

### Intervention

The Misfit Flash™ is a wearable activity tracker that measures step counts, tracks activity and estimates calories burned. The device can be worn with a wrist band or a clasp for attaching to shoes or clothing, depending on the activity type. It is water resistant up to 30 m and has a battery life of up to 6 months. The Misfit Flash™ synchronizes with compatible smartphones to provide feedback on activity levels and progress towards daily activity goals.

Participants and their parents were educated on the general use of the Misfit Flash™ through verbal and written information at the first clinic visit. A member of the research team offered assistance as needed with synchronizing the Misfit Flash™ to the participant’s smartphone. Demographic information including date of birth, gender, height and weight was entered in the program by each participant. A daily activity goal was set by each participant without input from the research team. For example, a daily goal of “active” corresponds to 1000 Misfit Flash™ points and can be achieved with a variety of activities such as 30 min of running or 45 min of swimming.

Participants were asked to wear the Misfit Flash™ for 24 h per day, 7 days a week for at least 28 consecutive days following the telephone interview 1 week after study enrollment. Participants were asked to return the device at the final study visit.

### Statistical analysis

Using descriptive statistics, the feasibility was measured by our ability to reach our desired sample size of 30 participants, as well as the proportion of those participants completing the study measurements and returning the device at study completion. Adherence with the activity tracker was assessed as the proportion of days with missing or incomplete data for each participant. The rate of device malfunction was also calculated.

Standardized response means were determined as a measure of responsiveness of the outcome variables to clinical change. The standard deviation of the paired score change was determined as a measure of variability. The standardized response mean was calculated as the difference in the mean values of self-reported 30-min blocks of MVPA and total METs per day from week one to week five divided by the standard deviation of the paired score change. The standardized response mean of the difference in the mean values of participant PROMIS® scores, pain scale, CHAQ and active joint count from baseline to week five were also determined with standard deviations calculated. A Bayes factor favouring the alternative hypothesis over the null hypothesis of no change (BF_10_) was used as a test of significance with a percent error calculated. To rule in favour of the alternative hypothesis, a BF_10_ ≥ 3 is considered significant.

## Results

Thirty-one patients met inclusion criteria and were willing to participate in the study. Of the 31 participants in the study, 28 were included in the estimation of effect size. One participant was diagnosed with inflammatory bowel disease during the study period and two participants withdrew due to school and extra-curricular commitments. Baseline characteristics for the participants included in the analysis are displayed in Table 2.

**Table 2:** Baseline characteristics for study participants (*n* = 31)

	Study Participants (*n* = 31)
Age in years, median (range)	15.1 (12.8–18.6)
Female (%)	23 (74)
JIA Subtype, n (%)	
Oligoarticular	7 (22.6)
Polyarticular (RF^a^ positive)	11 (35.5)
Polyarticular (RF^a^ negative)	4 (12.9)
Enthesitis Related	2 (6.5)
Psoriatic	4 (12.9)
Systemic	3 (9.7)
Medications, n (%)	
NSAID^a^	9 (29)
Prednisone	1 (3.2)
Non-biologic DMARD^a^	17 (54.9)
Biologic DMARD^a^	16 (51.6)

^a^*RF* rheumatoid factor, *NSAID* non-steroidal anti-inflammatory drug, *DMARD* Disease-modifying antirheumatic drugs

Values are the number of study participants (percent of sample size) unless otherwise specified.

### Feasibility

All of the participants were able to synchronize the Misfit Flash™ to a supported device, use the Misfit Flash™ correctly and complete the study measurements. Twenty-six participants had usable wear time data. One participant lost the device and another lost smart phone privileges and was unable to access the application. On average, participants had activity logged on their Misfit Flash™ application for 72% of the days in the intervention period. Five participants (19%) wore their Misfit Flash™ every day of the study period. Fifteen participants (58%) wore the Misfit Flash™ for at least 80% of the days of the study period. The majority of participants (55%) returned the Misfit Flash™ at the completion of the study.

The device malfunction rate for the Misfit Flash™ was 53%. Eight participants (29%) reported the battery died and their device stopped working during the study period. Four participant’s devices stopped working after wearing them in the water and three participants reported that the activity tracker disc fell out of the wrist band. Despite the technical issues, all but one participant (96%) reported that they enjoyed using the Misfit Flash™ and 89% were interested in continuing to use an activity tracker after completing the study.

Nine participants reported that illness, injury or pain prevented them from being active at some point in the study period. One patient had arthritis-related knee and ankle pain in the last week of the study period.

### Activity tracker and physical activity level

**Fig. 1:**
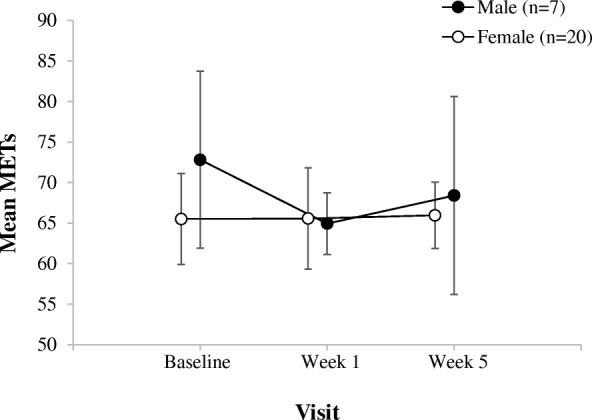
Sex differences of the change in mean METs from baseline visit to week 5. Line graph shows the mean METs/day and 95% confidence intervals (95% CIs) corresponding to each visit time

**Fig. 2:**
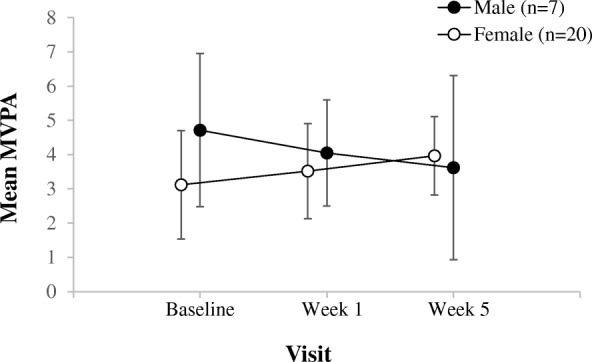
Sex differences of the change in mean MVPA values from baseline visit to week 5. Line graph shows the mean MVPA blocks/day and 95% confidence intervals (95% CIs) corresponding to each visit time

There was no significant difference in the mean METs/day and mean number of MVPA blocks/day from week 1 to week 5 (Table 3). The standard deviation of the change in METs/day was 12.148 and for MVPA blocks/day was 3.143 over the study period (Table 3). Bayesian repeated measures ANOVA, showed no effect of sex on the change in METs/day and number of MVPA blocks/day over time (Figs. 1 and 2).

**Table 3:** Changes from baseline in mean values of study variables and clinical parameters

Measure	Mean_baseline_	Mean_week5_	SD_baseline_	SD_week5_	SRM	SD_change_	BF_10_	%error
MET^a^	65.728	66.710	12.270	11.090	0.135	12.148	0.253	0.012
MVPA^a^	3.722	3.905	3.045	2.787	0.114	3.143	0.237	0.009
CHAQ	0.333	0.392	0.679	0.639	−0.090	0.299	0.250	0.022
Pain^b^	1.319	1.890	2.171	2.550	0.281	2.523	0.463	0.012
Swollen Joint Count	0.310	0.500	0.541	1.051	0.287	0. 693	0.476	0.012
Physician Global Assessment	0.429	0.711	1.016	1.217	0.355	0.604	1.025	0.003
Fatigue^c^	46.400	43.805	12.791	14.417	0.177	17.001	0.257	0.021
Depression^c^	46.570	43.160	9.118	8.328	− 0.039	10.385	0.468	0.018
Pain Interference^c^	45.200	46.336	10.706	11.132	0.332	11.860	0.522	0.015

*SD* standard deviation, *SRM* standardized response mean, *BF*_*10*_ Bayes factor favouring the alternative hypothesis over the null hypothesis of no change, *%error* percent error

^a^The mean and SD values from week 1 were used instead of the baseline visit for METs and MVPA

^b^Measured on a 10-cm visual analog scale

^c^Measured using the Patient Reported Outcomes Measurement Information System

The majority of participants (71%) reported that they felt like they were more physically active as a result of the Misfit Flash™ when asked at the completion of the study.

### Activity tracker and self-reported symptoms and clinical parameters

There was no significant difference in the mean PROMIS® scores from the baseline visit to week five (Table 3). There was also no significant difference in the mean CHAQ disability index, pain scale or active joint count from the baseline visit to week five (Table 3).

## Discussion

Overall, the Misfit Flash™ wearable activity tracker is a feasible intervention for adolescents with JIA. While we have estimated the variability of the change in physical activity parameters for use in planning a definitive trial, device malfunction issues should be addressed beforehand.

As a result of a short recruitment window and a convenience sample, the oligoarticular JIA subtype was under-represented in our sample. There was also a high proportion of patients receiving both non-biologic and biologic disease modifying anti-rheumatic drugs. Although we sampled from all of the JIA clinics including subspecialty (i.e. juvenile spondyloarthropathy and systemic JIA) clinics, young people with ERA were under-represented in our sample. With no established difference in physical activity levels based on JIA subtype or disease activity level [8], we believe that these factors do not materially affect our physical activity results or the generalizability of our feasibility aims.

Two participants withdrew from the study due to school and extra-curricular commitments. As busy schedules and time demands are common aspects of adolescents’ lives, ensuring that participants have adequate time and energy to engage in the intervention will be a challenge for future studies that other researchers should note and anticipate. Involving adolescents in the study design process could help address this and other potential issues for this age group.

In our review of the literature, we found one similar feasibility study that used a 2-week FitBit® intervention in 17 pediatric patients with acute lymphoblastic leukemia during the maintenance phase of their therapy [24]. They also reported a high proportion (94%) of participants that were able to synchronize the FitBit® and complete the study measurements. The superior wear time reported in their study (i.e., 92% vs. 72% of days) may be explained by the shorter intervention period. In contrast to our study, they did not report any FitBit® malfunction. This could also be related to the shorter intervention period or differences in device quality. The FitBit® devices are much more expensive than the comparable Misfit™ models, which may explain differences in malfunction rates but is an important factor in determining their utility in clinical and research settings.

Despite high malfunction rates, we received positive feedback from the participants during their study exit interviews. Almost all participants reported that they enjoyed wearing the tracker and the majority expressed interest in purchasing a tracker at the completion of the study. This positive feedback and interest in continued use demonstrates that activity trackers are well accepted in this population and supports the need for further research to determine their effectiveness in increasing physical activity levels. Piloting the feasibility questionnaire with adolescents beforehand could provide additional insight to improve the quality of feedback obtained from the participants in future studies.

The device return rate (55%) was low in our study. As many of the unreturned devices had malfunction, we did not make any additional attempts to have them returned if participants did not bring them to the final visit. Implementing better controls over returning the devices and removing barriers to returning them (e.g, postage paid return envelopes, reminder calls prior to the final study visit, etc.) would help address this issue in future studies.

The mean number of MVPA blocks per day and mean total METs per day for our sample were similar to those previously reported in healthy adolescent samples [4, 5]. Given that children and adolescents with JIA are less active than age-matched controls [8–10], there may have been a selection bias towards participants that are more physically active at baseline than the average JIA patient. Our result is even more surprising when we consider that female adolescents are less active than males [7] and 74% of our sample was female. Obtaining more detailed recruitment data including the reasons participants declined could help address this issue in future research. Alternatively, participants may have over-reported activity levels as a result of a social desirability bias. Finally, we tried to minimize the influence of the Hawthorne effect on the self-reported pre- and post-intervention PA levels by using the 3DPAR completed 1-week after study enrollment (but prior to using the activity tracker) as a baseline so that participants knew they were being observed for both the pre- and post-intervention measures of PA. While including more motivated patients could skew our feasibility results, we don’t anticipate an effect on our measures of variability in the change of physical activity parameters as a result.

While there was no difference in pre- and post-intervention physical activity levels in our study, Hooke et al. report a trend towards increased steps per day from week 1 and week 2 with their FitBit® intervention [24]. Participant steps per day as a measure is more sensitive to change than blocks MVPA per day and total METs per day. Their intervention also incorporated daily coaching emails from a study nurse which may have an independent effect on participant activity levels. Future studies that assess these interventions separately may be needed to tease out the independent effect of the activity tracker on activity levels. Ideally, further research would also examine the sustainability of an effect with a study design that includes long-term follow up.

Although there was no significant difference in their 3DPAR scores, most participants reported a subjective increase in their activity level as a result of wearing the activity tracker. Since participants only wore the trackers while they were functional, we hypothesize that the high malfunction rates limited the effect of the intervention on physical activity levels.

There was no significant difference in participants PROMIS® scores or clinical parameters with our intervention. While there were few studies to compare with, Hook et al. also report no change in self-reported fatigue with their 2-week FitBit® intervention.

As there is no current evidence to support an effect of exercise intervention on JIA disease activity from previous studies, it is not surprising that this short intervention found no significant difference in clinical parameters. Based on our reported variability of change in physical activity parameters, our study was likely too small to have detected a difference should one exist.

Finally, the device we chose, primarily due to funding constraints, may have contributed to high malfunction rates. This issue should be considered in future research.

## Conclusion

Overall, mobile activity trackers are feasible to employ and study and are well-tolerated and enjoyable for adolescents with JIA. We have estimated the variability in activity and response to wearing the tracker that will allow for the planning of a definitive intervention trial.

## Abbreviations

3DPAR: 3-Day Physical Activity Recall
BF_10_: Bayes factor favouring the alternative hypothesis over the null hypothesis of no change
CHAQ: Child Health Assessment Questionnaire
JIA: Juvenile idiopathic arthritis
METs: Metabolic equivalents
MVPA: Moderate-to-vigorous physical activity
RF: Rheumatoid factor
